# Supplementary material for: Extracellular Vesicles Released by Colorectal Cancer Cell Lines Modulate Innate Immune Response in Zebrafish Model: The Possible Role of Human Endogenous Retroviruses
Source: Int J Mol Sci. 2019 Jul 26;20(15):3669. doi: 10.3390/ijms20153669 (PMC6695895; doi:10.3390/ijms20153669)

Supplementary materials and methods

MISEV 2018 guidelines compliance

	Section title	Required information according to MISEV2018	Mandatory requirement	Not applicable/not available	Our approach	Compliance with MISEV2018 requirements
1	Nomenclature	The term extracellular vesicle (EV) can be used with demonstration of extracellular (no intact cells) and vesicular nature per these characterization and function	YES		As explained in section 4 and 5, the term extracellular vesicle (EV) has been used in the manuscript	YES
2a	Collection and pre-processing (tissue culture conditioned medium)	General cell characterization	YES		-Human Caco-2 cell line (ATCC® HTB-37, Basel, Switzerland) derived from colorectal adenocarcinoma of a 72-year old Caucasian male patient.<br>-SK-CO1 (ATCC® HTB-39, Basel, Switzerland) cell line was derived from the metastatic site ascites, from a colorectal adenocarcinoma of a 65-year old Caucasian male patient.<br>- Cells were mycoplasma free.	YES
2a	Collection and pre-processing (tissue culture conditioned medium)	Medium used before and during collection (additives, serum, other)	YES		-Caco-2 cell line was grown and sub-cultured in DMEM medium (Sigma-Aldrich, Germany), containing L-glutamine (Sigma-Aldrich, Germany), 10% v/v fetal bovine serum and antibiotics	YES

					(50 U/mL penicillin; 50µg/mL streptomycin; Euroclone, Italy).<br>-SK-CO-1 cell line was grown and sub-cultured in EMEM medium (Sigma-Aldrich, Germany), containing L-glutamine (Sigma-Aldrich, Germany), 10% v/v fetal bovine serum and antibiotics (50 U/mL penicillin; 50µg/mL streptomycin; Euroclone, Italy).<br>-Cells were maintained at 37 °C in a 5% CO_2 humidified atmosphere.	
2a	Collection and pre-processing (tissue culture conditioned medium)	Exact protocol for depletion of EVs from additives in collection medium	YES		EVs were obtained after 24 hours cell culture in culture medium conditioned by cells in the absence of fetal calf serum.	YES
2a	Collection and pre-processing (tissue culture conditioned medium)	Nature and size of culture vessels, and volume of medium during conditioning e) specific culture conditions (treatment, % O_2, coating,polarization…) before and during collection	YES		Cells were cultured in T75 vented flask (Primo® TC Flask 75cm2 screw cap w/filter ET7076, Euroclone, Italy).<br>10 ml of medium were used for each flask.<br>Decitabine (5-Aza-2′-deoxycytidine, Sigma-Aldrich) treatments were performed at final concentration of 5µM, 96 hours before harvesting the cells.	YES
2a	Collection and pre-processing (tissue culture	Number of cells/ml and % of live/ dead cells at time of collection	YES		75000 cells/ml were seeded and % of dead cells at time of collection was below 10%.	YES

	conditioned medium)					
2a	Collection and pre-processing (tissue culture conditioned medium)	Frequency and interval of Conditioned Medium harvest	YES		EVs were obtained from culture medium conditioned by cells for 24 hours (from the end of 96 hours of treatment).	YES
2b and 2c	Collection and pre-processing (Buofluids or tissues)			N/A		
2d	Storage and recovery	Storage and recovery (e.g., thawing) of CCM, biofluid, or tissue before EV isolation (storage temperature, vessel, time; method of thawing or other sample preparation)	YES		Culture medium were processed within 1 hour from harvesting and kept at room temperature.	YES
2d	Storage and recovery	Storage and recovery of EVs after isolation (temperature, vessel, time, additive(s)…)	YES		Isolated EVs were immediately characterized by flow cytometry and Nanosight. Zebrafish injection was performed within 24 hours from medium harvesting.	YES
3	EV separation and concentration	Centrifugation: reference number of tube(s), rotor(s), adjusted k factor(s) of each centrifugation step (= time+ speed+ rotor, volume/density of centrifugation conditions),	YES		We applied a differential ultracentrifugation with previous lower-speed steps. 8 mL of medium were collected in Corning 15 mL PP Centrifuge Tubes, and centrifuged at 1000, 2000, and 3000 × g for 15 min at 4 °C (Haereus Labofuge 400R, Hanau, Germany). The obtained pellets were	YES

		temperature, brake settings			discarded to remove cells, apoptotic bodies and cell debris. EVs were then isolated from supernatants by ultracentrifugation at 110,000 × g for 4 hours at 4 °C in polypropylene ultracentrifuge tubes (Quick-Seal ultra-clear centrifuge tubes, Beckman Coulter; Brea, CA, USA) rotor MLA-55 (Beckman Coulter), filled with PBS previously filtered through a 0.10-μm pore-size polyethersulfone filter (StericupRVP, Merck Millipore; Burlington, MA, USA). Our method is included in the category "Intermediate recovery, intermediate specificity = mixed EVs with limited non-EV components"	
3	EV separation and concentration	Density gradient: nature of matrix, method of generating gradient, reference (and size) of tubes, centrifugation speed and time (with brake specified), method and volume of fraction recovery.		N/A		
3	EV separation and concentration	Chromatography: matrix (nature, pore size,…), loaded sample volume, fraction volume, number		N/A		

3	EV separation and concentration	Precipitation: reference of polymer, ratio vol/vol or weight/vol polymer/fluid, time/temperature of incubation, time/speed/temperature of centrifugation		N/A		
3	EV separation and concentration	Filtration: reference of filter type (=nature of membrane, pore size…), time and speed of centrifugation, volume before/after (in case of concentration)		N/A		
3	EV separation and concentration	Antibody-based : reference of antibodies, mass Ab/amount of EVs, nature of Ab carrier (bead, surface) and amount of Ab/carrier surface		N/A		
3	EV separation and concentration	Other…: all necessary details to allow replication		N/A		
3	EV separation and concentration	Additional step(s) to concentrate, if any		N/A		
3	EV separation and concentration	Additional step(s) to wash matrix and/or sample, if any		N/A		
4a	EV characterization, Quantification	Volume of fluid, and/or cell number, and/or tissue mass used to isolate EVs	YES		8 ml of culture medium were processed as explained in section 3 and the EV pellet was resuspended in 500 µl of triple-filtered PBS.	YES

4a	EV characterization, Quantification	Global quantification by at least 2 methods: protein amount, particle number, lipid amount, expressed per volume of initial fluid or number of producing cells/mass of tissue	YES		In order to quantify the total number of EVs we applied the two following approaches:<br>1) Nanoparticle tracking analysis by NanoSight NS300 system (Malvern Panalytical Ltd, Malvern, UK). Five 30-s recordings were made for each sample. Collected data were analyzed with NTA software (Malvern Panalytical Ltd.), which provided high-resolution particle-size distribution profiles as well as measurements of the EV concentration.<br>2) High resolution Flow cytometry by MACSQuant, Miltenyi Biotec. In order to analyze EV integrity, 60 µl aliquots were stained with 0.2 µM 5(6)-carboxyfluorescein diacetate N-succinimidyl ester (CFSE) at 37 °C for 20 min in the dark. CFSE is a cell permeant, non-fluorescent pro-dye. If incorporated into intact EVs, which contain esterases as live cells, the acetate groups of CFSE is cleaved producing a membrane-impermeant molecule with green fluorescence.	YES

4a	EV characterization, Quantification	Ratio of the 2 quantification figures	YES	N/A		
4b	EV characterization, General Characterization	At least three positive protein markers of EVs, including at least one transmembrane/lipid bound protein and one cytosolic protein<br><br>At least one negative protein marker	YES		The following antibodies have been used to measure positive protein markers of EVs:<br>-anti-CD63-APC (clone REA1055) (Miltenyi Biotec), transmembrane and non-tissue specific<br>-anti-CD326 (EpCAM)-APC (clone HEA-125) (Miltenyi Biotec), transmembrane and tissue specific<br>-anti-HERV-K (clone 5i73) (US Biological, MA, USA) transmembrane and focus of the present study<br>- anti-HERV-W (clone clone 4F10) (Sigma Aldrich) transmembrane and focus of the present study.<br><br>CFSE staining was used to assess cytosolic esterase activity. As negative control, representative EVs samples were incubated with Tween20 (P1379; Merk, Sigma-Aldrich) ON at 37 °C, and then incubated with CFSE as described above. Positivity for CFSE fluorescence was evaluated by High Resolution Flow cytometry analysis	YES

4c	Single EV characterization	Images of single EVs by electron microscopy	YES		Transmission Electron Microscopy (TEM) analysis was performed on random samples as quality control.	YES
4c	Single EV characterization	Non-image-based method analysing large numbers of single EVs: Non-image-based method analysing large numbers of single EVs: NTA, TRPS, FCS, high-resolution flow cytometry, multi-angle light-scattering, Raman spectroscopy, etc.	YES		NTA and , high-resolution flow cytometry were performed	YES
5	Functional studies	Dose-response assessment	YES		EVs were normalized by volume and injected in zebrafish embryos at their original concentration. The number of (HERV-positive) injected EV was then associated to the functional effect (i.e. innate immune response)	YES
5	Functional studies	Negative control = nonconditioned medium, biofluid/tissue from control donors, as applicable	YES		Nonconditioned medium was injected as negative control.	YES
5	Functional studies	Quantitative comparison of functional activity of total fluid, vs EV-depleted fluid, vs EVs	YES		Functional effects were evaluated by real-time quantitative PCR.	YES

		(after high recovery/low specificity separation)				
5	Functional studies	Quantitative comparison of functional activity of EVs vs other EPs/fractions after low recovery/high specificity separation	YES	N/A		
5	Functional studies	Quantitative comparison of activity of EV subtypes (if subtype-specific function claimed)	YES	N/A		
5	Functional studies	Extent of functional activity in the absence of contact between EV donor and EV recipient	YES	N/A		
6	Reporting	Submission of data (proteomic, sequencing, other) to relevant public, curated databases or open-access repository	YES	N/A		

Supplementary methods for High Resolution Flow Citometry

Microvesicles have been isolated from blood samples within four hours after blood drawing.

Preparation of microvesicles from cells:

1. Draw 8 mL of cell medium into 15 mL tubes.

2. Separate plasma by centrifugation at 1,100×g for 15 minutes at room temperature.

3. Remove cell debris from medium by serial centrifugation at 1,000, 2,000, and 3,000×g for 15 minutes at 4 °C.

4. Transfer 5 mL of supernatant into an ultracentrifuge tube and fill up with 0.10 µm pore size membrane-filtered PBS.

5. Ultracentrifuge sample at 110,000×g for 75 minutes at 4 °C.

6. Resuspend the ultracentrifuged pellet with 500 µL triple 0.10 µm pore size membrane-filtered PBS.

7. Transmission electron microscopy (TEM) was used to check the morphology of microvesicles and aggregates.

8. 100 µL of unstained sample were used for NanoSight analysis to check for aggregates and the size and concentration of microvesicles.

Staining of sample

9. 60 µL of sample and 60 µL of triple 0.10 µm pore size membrane-filtered PBS (control sample) were stained with 0.02 µM CFSE at 37 °C for 20 minutes in the dark.

10. The CFSE stained sample and the control sample were incubated with 6 µL of HERV-K, HERV-W, CD63, EpCAM antibodies in the dark for 20 minutes at 4 °C.

Note: Before use the antibodies were centrifuged at 17,000×g for 30 minutes at 4 °C to eliminate aggregates.

Data acquisition and analyzes using the MACSQuant Analyzer

Note: Sheath fluid was filtered using 0.1 µm pore size filter to further improve the signal-to-noise ratio.

11. The emission spectra were compensated to correct the spectral overlap (Figure 1).

12. Unstained triple 0.10 µm pore size membrane-filtered PBS was acquired to evaluate the buffer background noise.

13. The stained PBS control sample was acquired to detect the autofluorescence of the antibodies (Supplementary Figures S5-S8).

14. 30 µL of unstained sample were acquired to detect the sample auto-fluorescence (Supplementary Figures S5-S8).

15. The Fluoresbrite® Carboxylate Size Range Kit I (0.2, 0.5, 0.75, and 1 µm) was used to set the calibration gate in the FSC/FL1 and FSC/SSC dot plots on MACQSQuant Analyzer (Supplementary Figures S5-S8).

16. 30 µL of double stained sample were acquired on the MACSQuant Analyzer.

17. Quantitative multi-parameter analysis of flow cytometry data was carried out using FlowJo Software (Tree Star, Inc.) to determine the percentage and count of double stained microvesicles (CFSE and PE positive events).

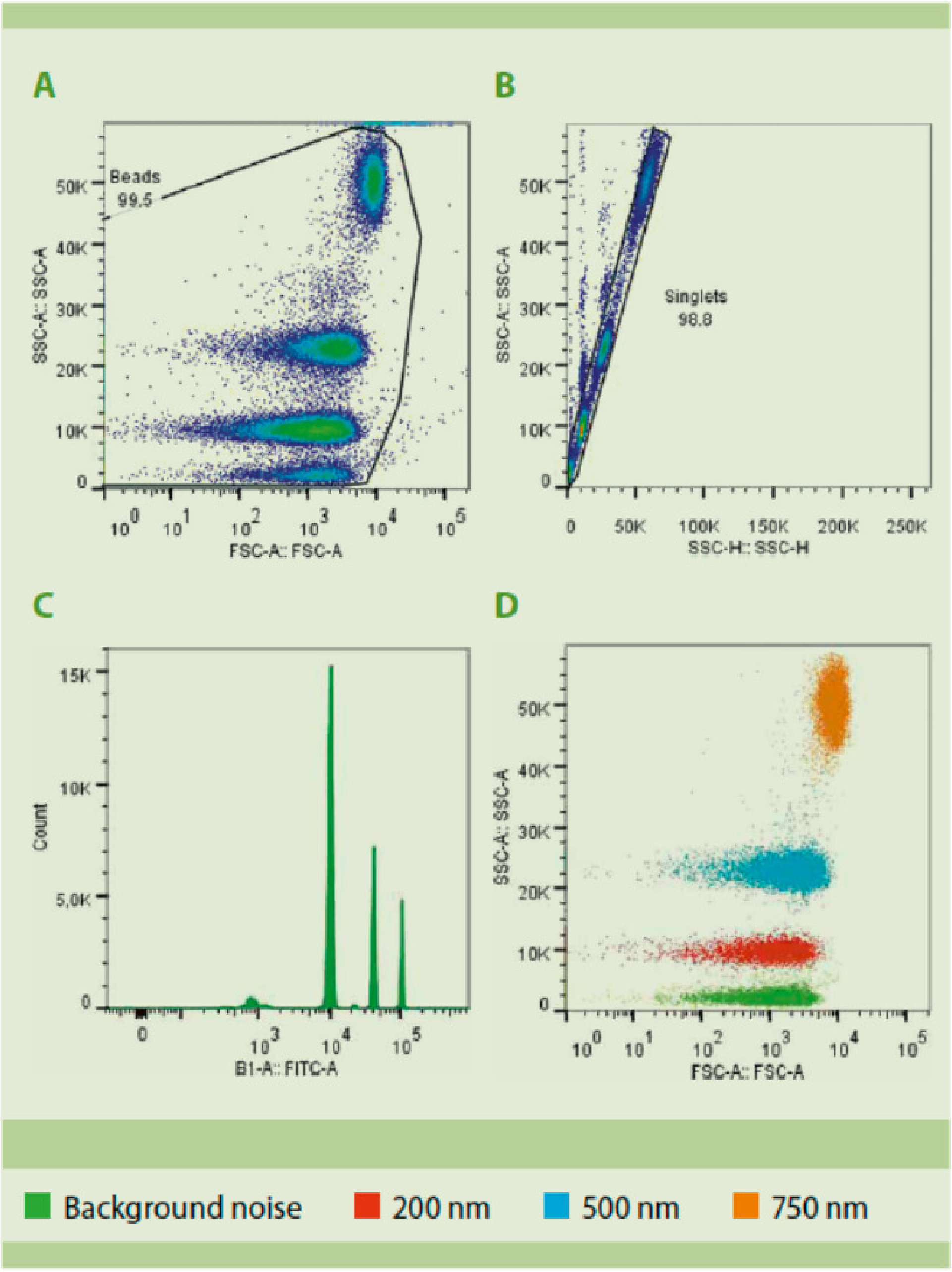

Figure 1: Scatter and fluorescence calibration: Fluoresbrite Carboxylate Size Range Kit was used to obtain optimal resolution of scatter and FITC signals. A) Debris exclusion, B) Doublet exclusion, C) Histogram showing resolution of the different bead populations according to FITC fluorescence, D) Resolution of the different bead populations according to FSC and SSC signals.

Supplementary Tables and Figures

Supplementary Table S1: Association of *HERVs* expression levels (RQ) between untreated- and decitabine-treated- Caco-2 and SK-CO-1 cell lines.

**Element**	**Trattamento**	**Mean**	**Standard Deviation**	**Median**	**Range interquartile**	**T-test**	**Wilcoxon**
**Caco-2 cells**							
HERV-K	Untreated	0.0331	0.0138	0.0260	0.0252	0.207	0.298
	Treated	0.0481	0.0254	0.0414	0.0202		
HERV-W	Untreated	0.0041	0.0044	0.0028	0.0033	0.093	0.095
	Treated	0.0155	0.0113	0.0126	0.0106		
HERV-H	Untreated	0.0006	0.0004	0.0005	0.0008	**<0.001**	0.061
	Treated	0.0055	0.0068	0.0026	0.0055		
HERV P	Untreated	0.0027	0.0018	0.0027	0.0024	0.099	0.575
	Treated	0.0049	0.0041	0.0035	0.0079		
**SK-CO-1 cells**							
**Element**	**Trattamento**	**Mean**	**Standard Deviation**	**Median**	**Range interquartile**	**T-test**	**Wilcoxon**
HERV-K	Untreated	0.00008	0.00003	0.00008	0.00005	**0.045**	0.112
	Treated	0.00013	0.00001	0.00013	0.00002		
HERV-W	Untreated	0.00096	0.00009	0.00096	0.00013	**0.011**	**0.030**
	Treated	0.00151	0.00028	0.00153	0.00047		
HERV-H	Untreated	0.00016	0.00004	0.00015	0.00006	**0.003**	**0.030**
	Treated	0.00040	0.00009	0.00040	0.00015		
HERV P	Untreated	0.00237	0.00064	0.00212	0.00076	**0.026**	**0.030**
	Treated	0.00368	0.00007	0.00368	0.00010		

Supplementary Table S2: Primers sequences.

PRIMERS	SEQUENCES	
**Methylation**	**Forward**	**Reverse**
HERV-K	5'-GTAAAGGGTTTGTGTTGAGGAG-3'	5'-BIO-ACTTATCCCACACCTCCAAC-3'
HERV-W	5'-ATGGAGTTTAAGATGTAGTTTAAG-3'	5'-BIO-CAATCCCCCATCTCAACAA-3'
HERV-H	5'-BIO-AAAAGGAGGAAAAGTAAAGAAAGA-3'	5'-CCAAAAAAAAAAATTTCACAAAA-3'
HERV-P	5'-BIO-TGTGGAGAAAGAAGTTTGATGTTA-3'	5'-CCTTTTAATCTCTTCACTAATT-3'
LINE-1	5'-TTTTGAGTTAGGTGTGGGATATA-3'	5'-BIO-AAAATCAAAAAATTCCCTTTC-3'
**Sequencing**		
HERV-H	5'-CAATTACTTCAAACCATCTA-3'	
HERV-K	5'-TTTTGGGTAATGGAATG-3'	
HERV-W	5'-AGTTTAAGATTAAGATTTAT-3'	
HERV-P	5'-CCCTTTAAATCACAACC-3'	
LINE-1	5'-AGTTAGGTGTGGGATATAGT-3'	
**Expression**	**Forward**	**Reverse**
GAPDH	5'-GCCCAGGATGCCCTTGA-3'	5'-GTGTCCCCACTGCCAAC-3'
B-ACTIN	5′- TGAGAGGGAAATCGTGCGTGAC-3′	5′- GCTCGTTGCCAATAGTGATGACC-3′
HERV-H	5'- TTCACTCCATCCTTGGCTAT-3'	5'- CGTCGAGTATCTACGAGCAAT-3'
HERV-K	5'-CACAACTAAAGAAGCTGACG-3'	5'-CATAGGCCCAGTTGGTATAG-3'
HERV-W	5'-TCATATCTAAGCCCCGCAAC-3'	5'-CGTTCCATGTCCCCATTTAG-3'
HERV-P	5- CAAGATTGGGTCCCCTCAC-3'	5'-CCTATGGGGTCTTTCCCTC-3'
IL1-β	5′-TGGACTTCGCAGCACAAAATG-3′	5′-CGTTCACTTCACGCTCTTGGATG-3′
mpx	5′-GCTGCTTACAAGTATTCTCG-3′	5′-ACGGCCTCCCGTGTCTTTCG-3′
IL-10	5′-TTGGAGACCATTCTGCCAACAGC-3′	5′-TGCATTTCACCATATCCCGCTTG-3′
rpl8	5′-CTCCGTCTTCAAAGCCCAT-3′	5′-TCCTTCACGATCCCCTTGAT-3′

Supplementary Figure S1: Spaghetti plot and box plot of *LINE-1* methylation levels (5%mC) in untreated and decitabine-treated Caco-2 and SK-CO1 cells.

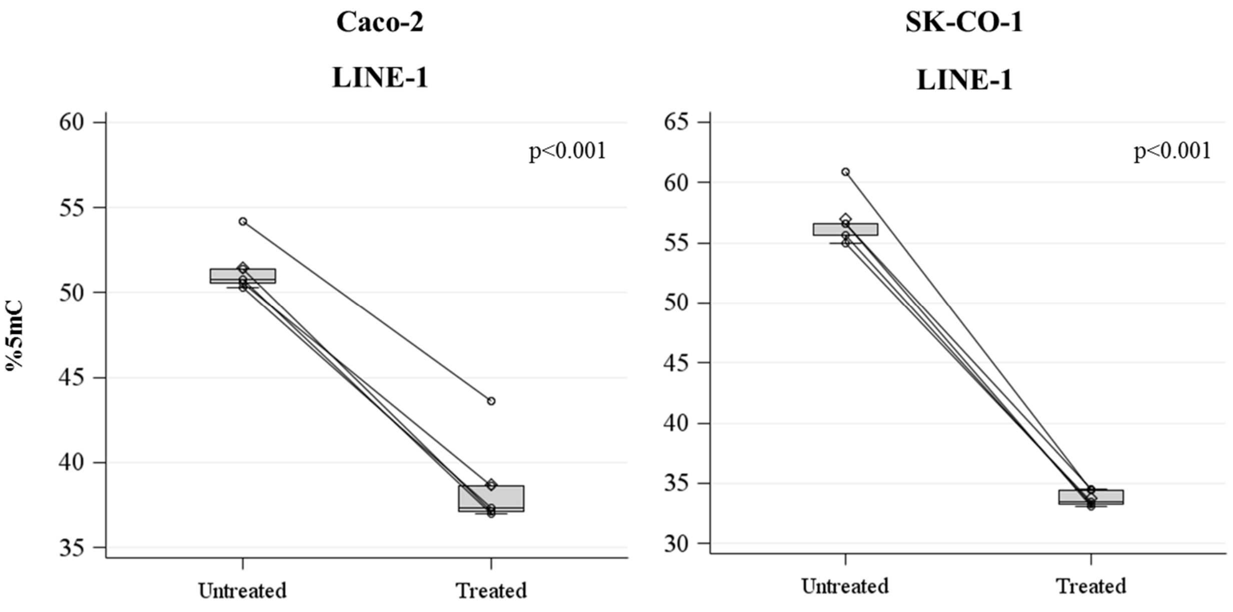

Supplementary Figure S2: HERV-W gating protocols for Caco-2 (A-D) and SK-CO-1 (E-H) cells. A, E: scatter plots; B, F: dot plots with gating for CFSE fluorescence; C, G: dot plots for experimental sample (scatter+ CFSE + ANTI-HERV-W antibody); D, H: dot plots for scatter+ ANTI-HERV-W antibody.

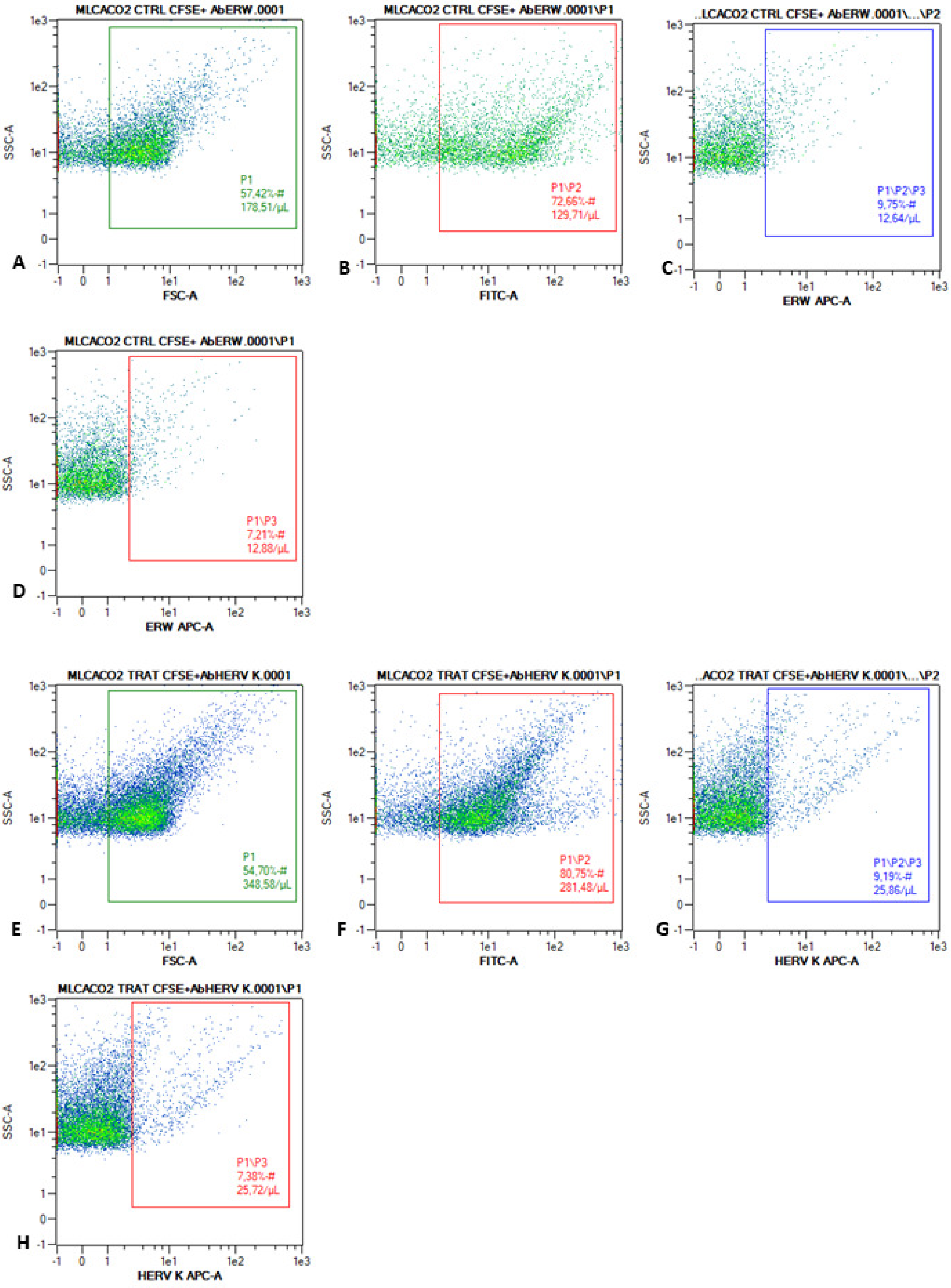

Supplementary Figure S3: HERV-K gating protocols for Caco-2 (A-D) and SK-CO-1 (E-H) cells. A, E: scatter plots; B, F: dot plots with gating for CFSE fluorescence; C, G: dot plots for experimental sample (scatter+ CFSE + ANTI-HERV-K antibody); D, H: dot plots for scatter+ ANTI-HERV-K antibody.

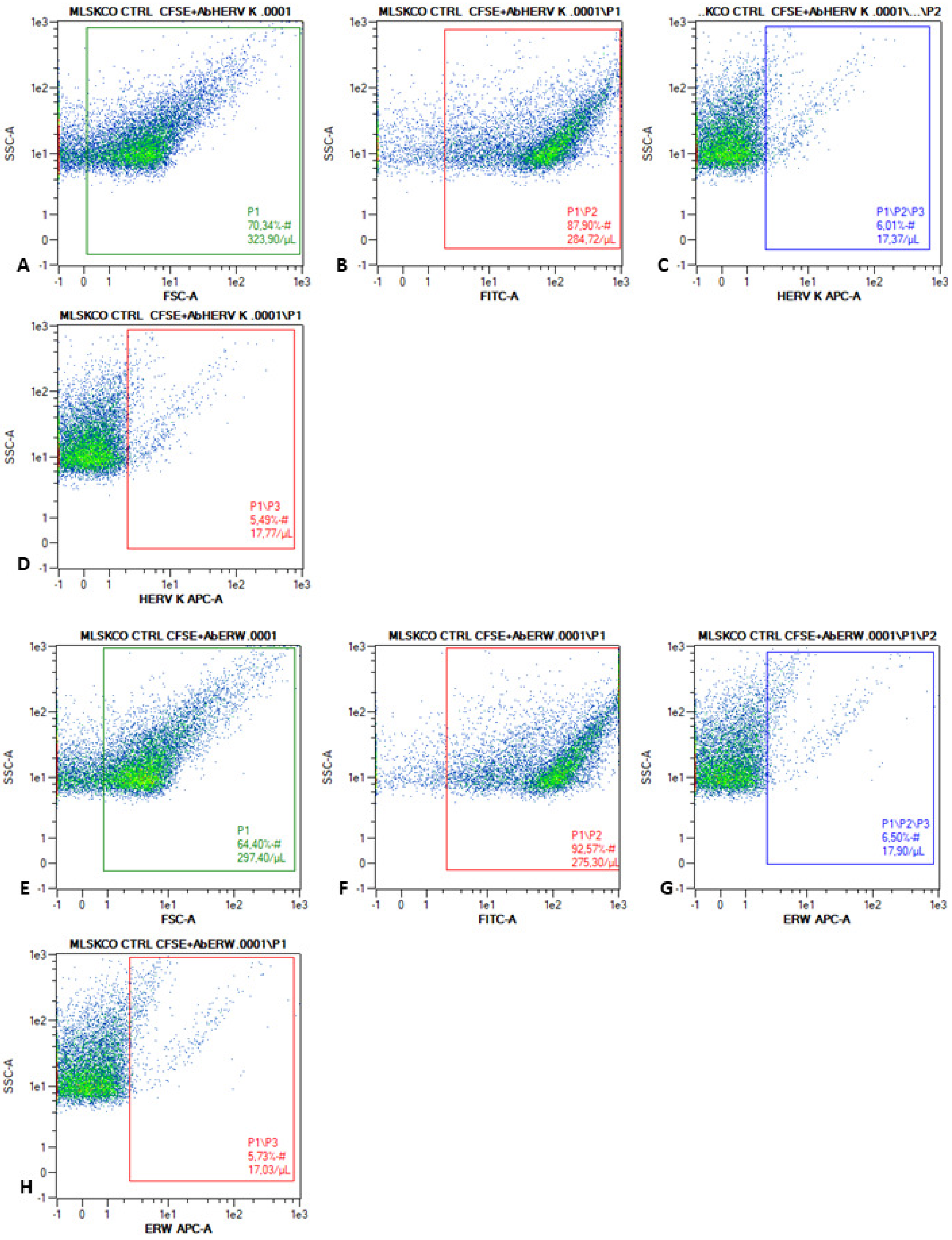

Supplementary Figure S4: Spaghetti plot and box plot of HERV-K and HERV-W positive EVs concentrations (count/mL) in untreated and decitabine-treated Caco-2 and SK-CO1 cells.

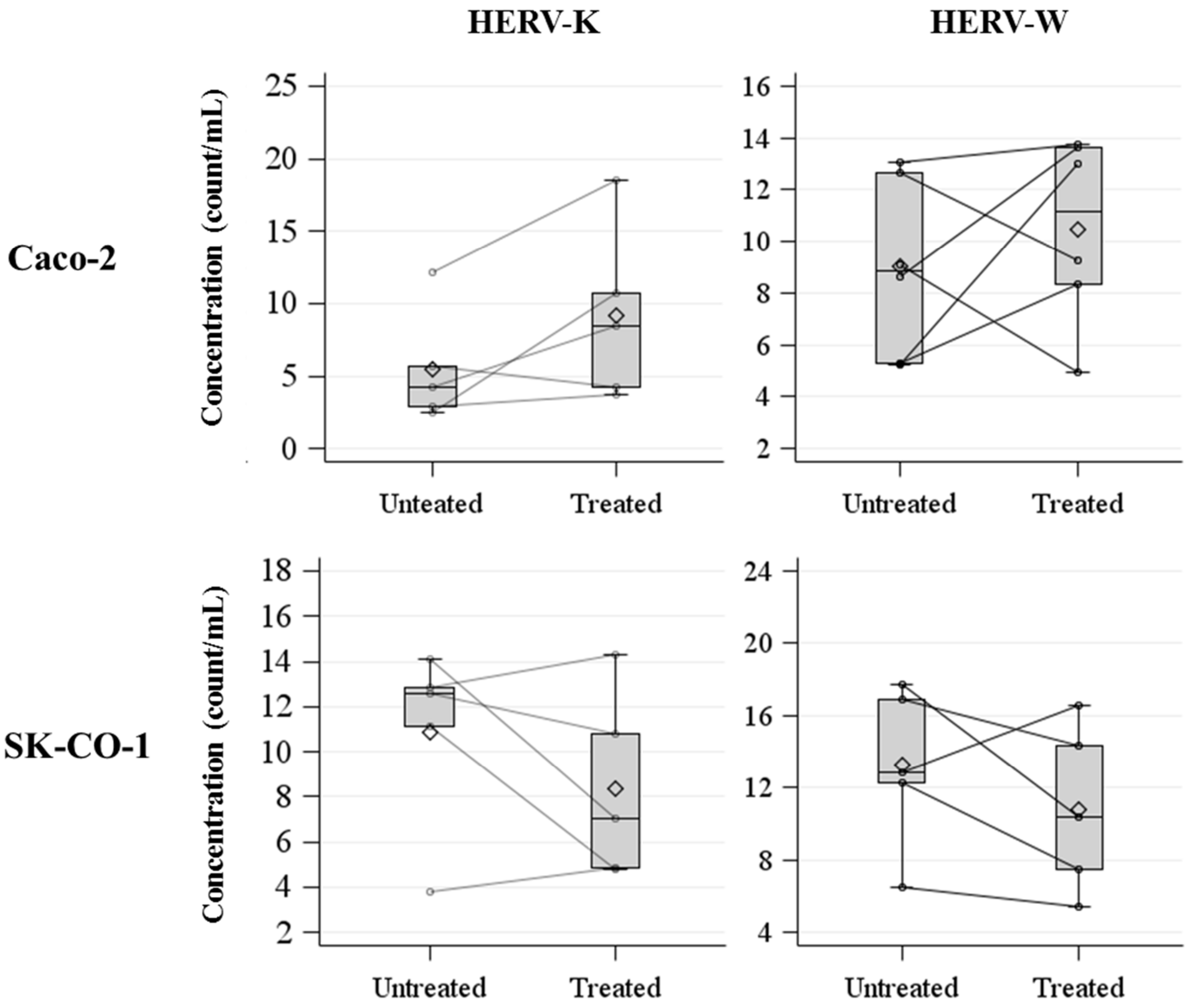

Supplementary Figure S5: Gating strategy and gating controls for anti-HERV-K staining for "background noise" control (Triple-filtered PBS only), Caco-2 cells, and SK-CO-1 cells. A-C: scatter (A), CFSE fluorescence (B), scatter+ CFSE + ANTI-HERV-K antibody (C).

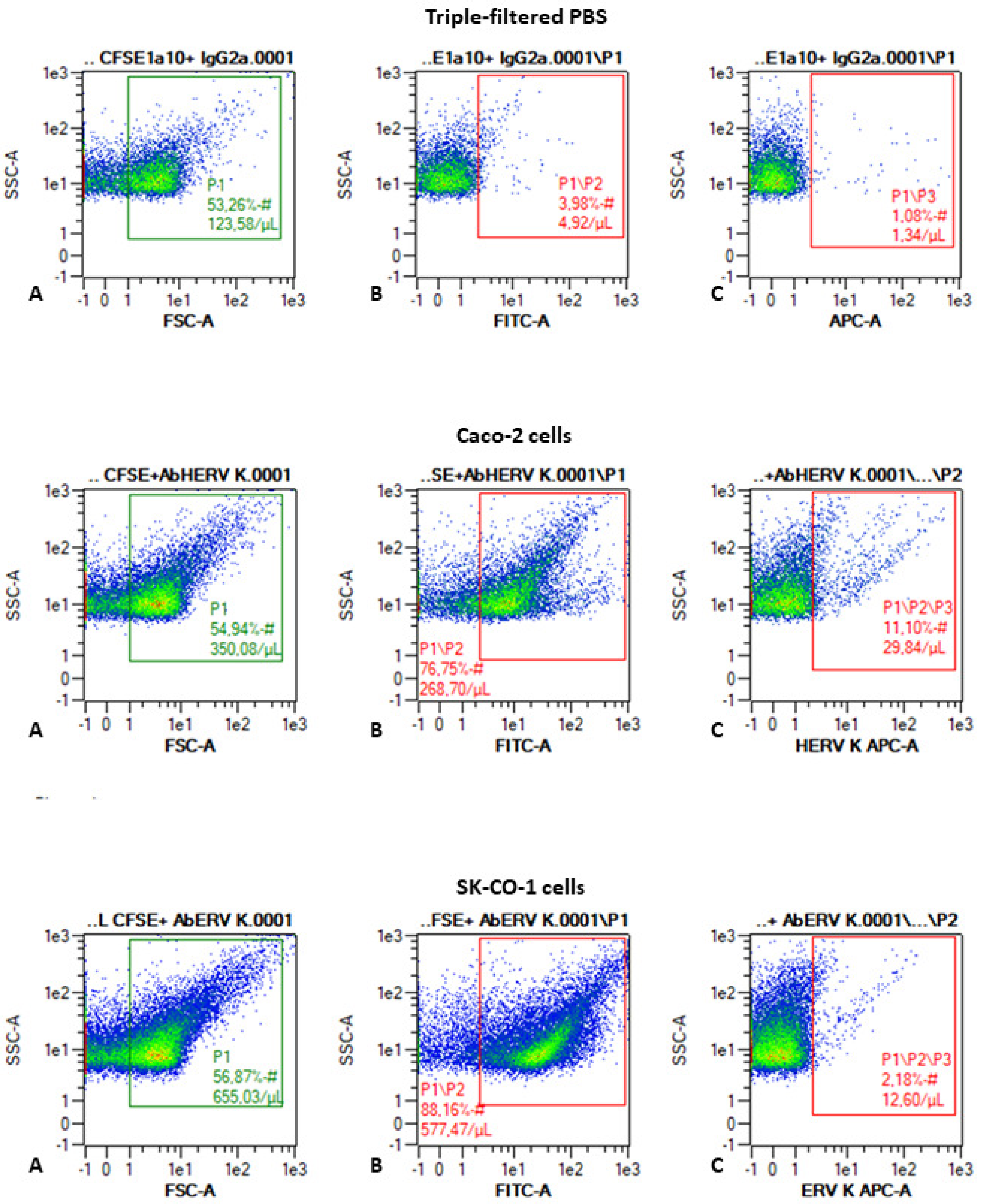

Supplementary Figure S6: Gating strategy and gating controls for anti-HERV-W staining for “background noise” control (Triple-filtered PBS only), Caco-2 cells, and SK-CO-1 cells. A-C: scatter (A), CFSE fluorescence (B), scatter+ CFSE + ANTI-HERV-W antibody (C).

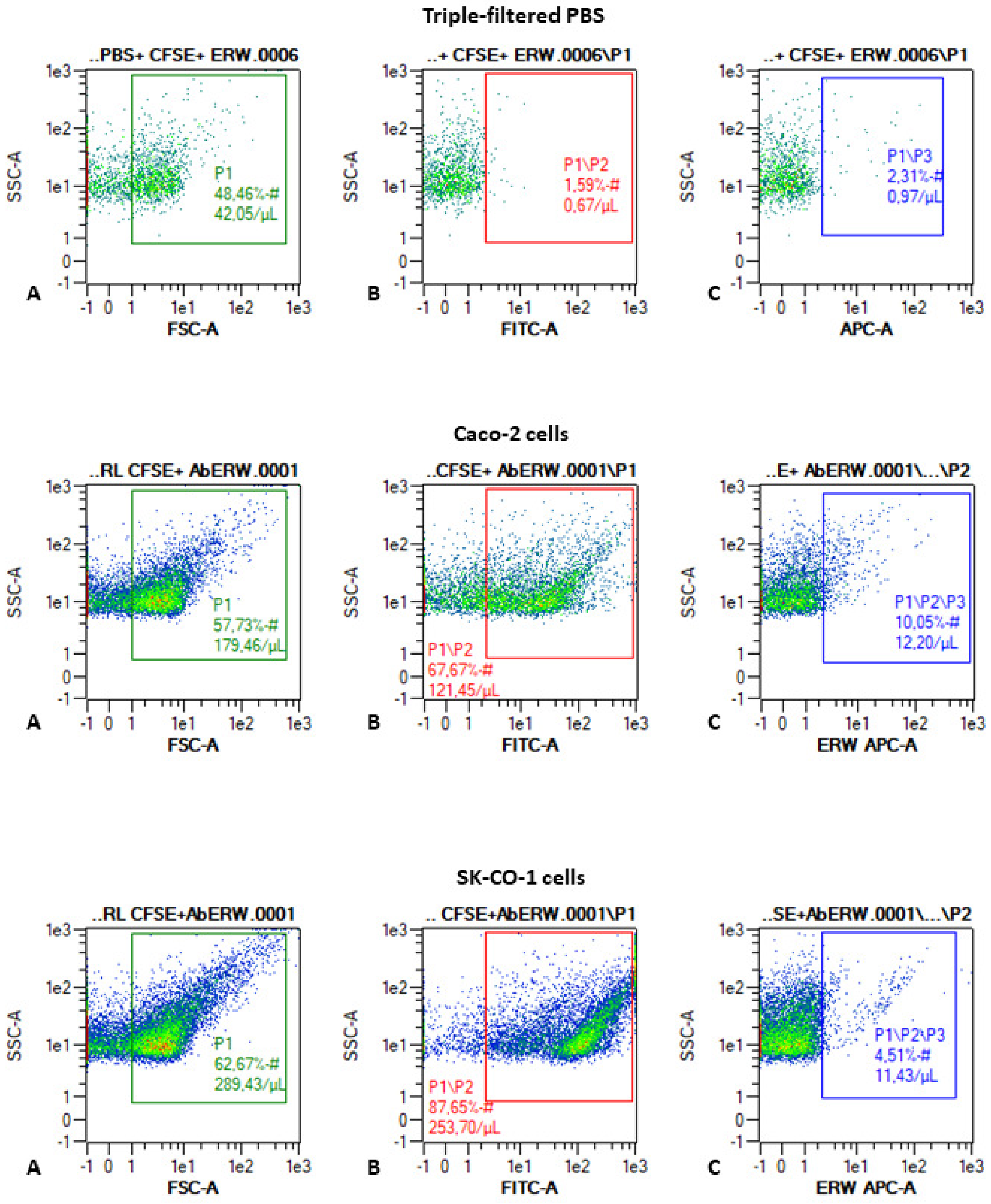

Supplementary Figure S7: Gating strategy and gating controls for anti-EPCAM staining for "background noise" control (Triple-filtered PBS only), Caco-2 cells, and SK-CO-1 cells. A-C: scatter (A), CFSE fluorescence (B), scatter+ CFSE + ANTI-EPCAM antibody (C).

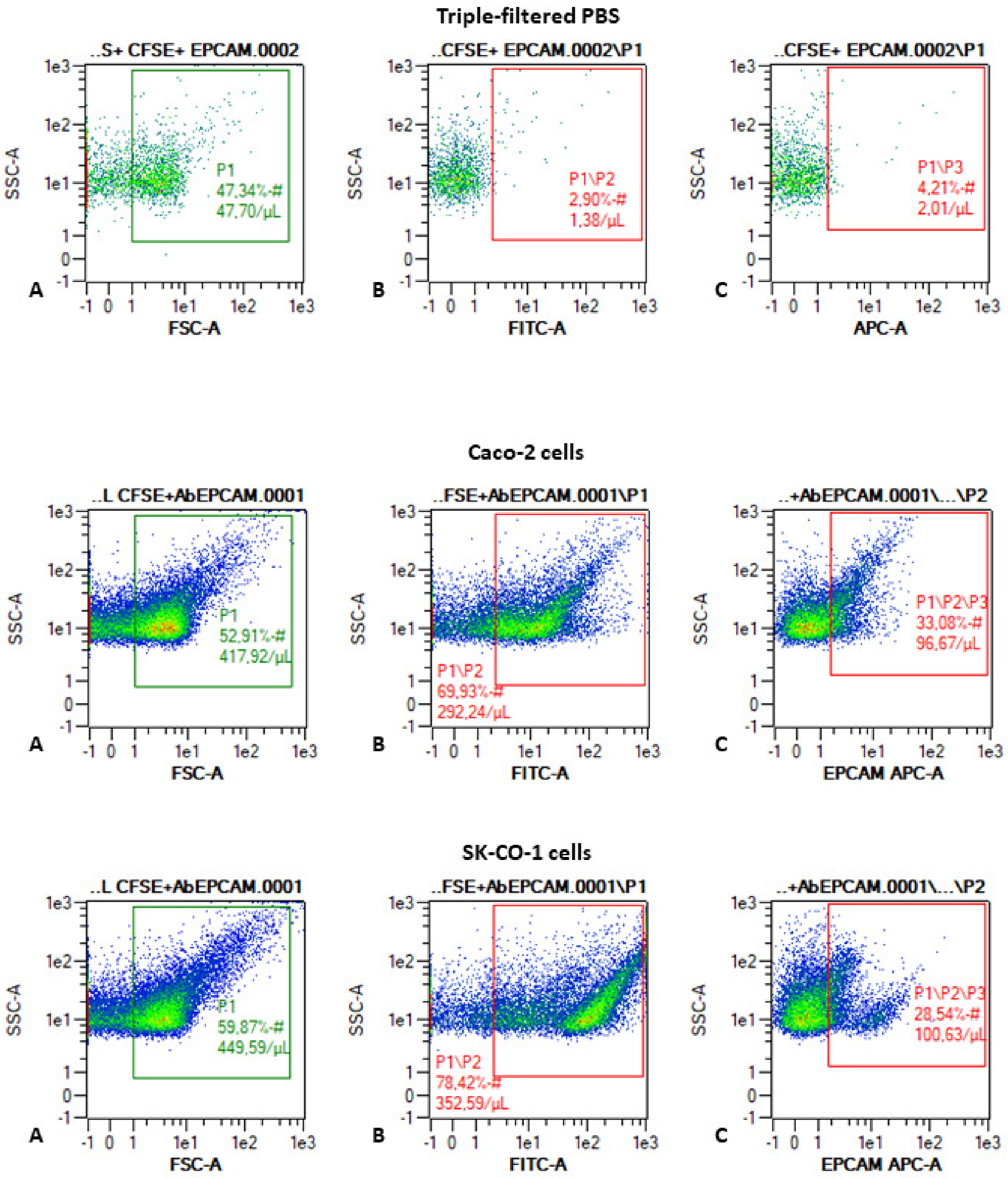

Supplementary Figure S8: Gating strategy and gating controls for anti-CD63 staining for "background noise" control (Triple-filtered PBS only), Caco-2 cells, and SK-CO-1 cells. A-C: scatter (A), CFSE fluorescence (B), scatter+ CFSE + ANTI-CD63 antibody (C).

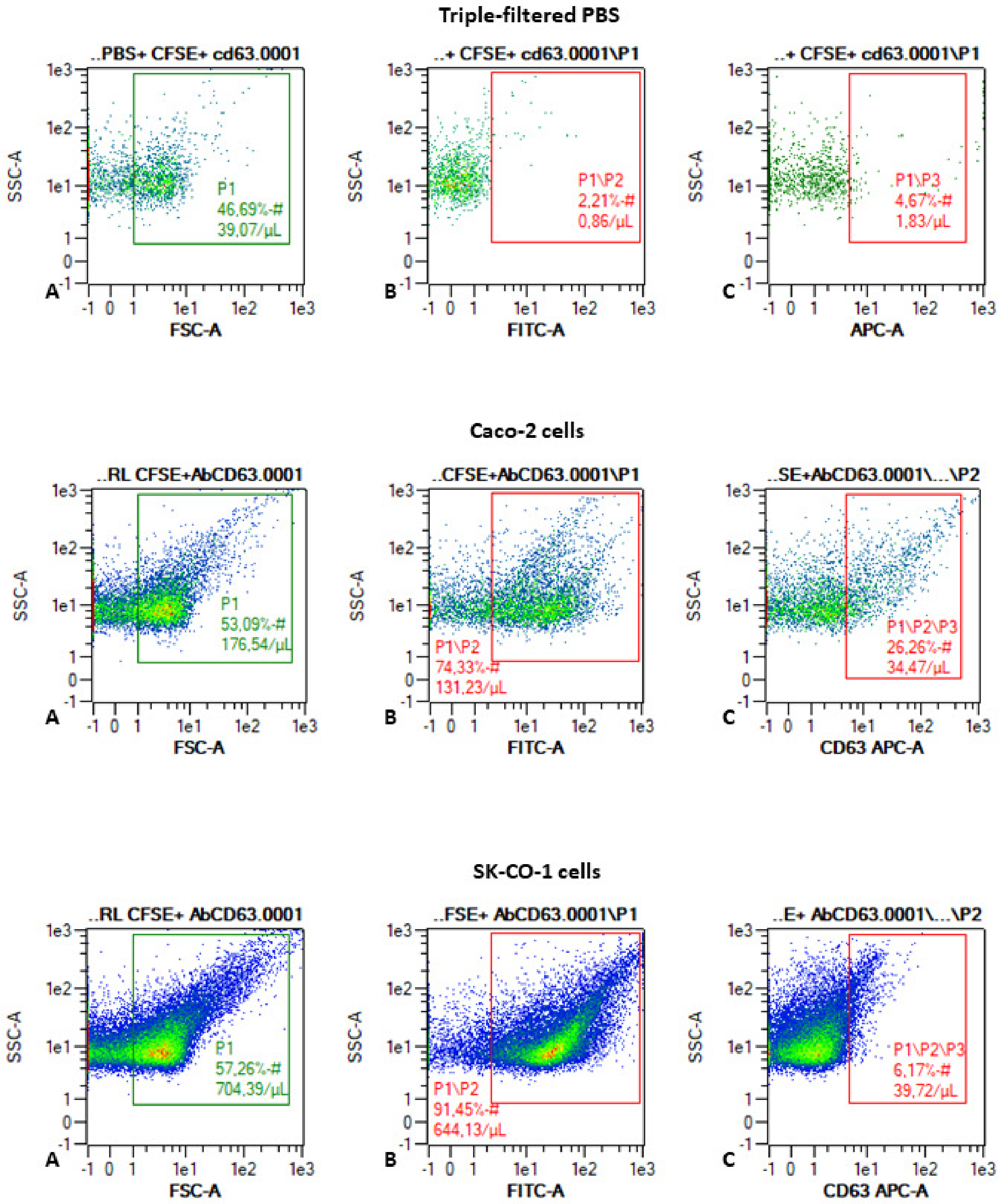

Supplementary Figure S9: Validation of CFSE staining Specificity. A-C: scatter plots EVs (A); scatter plots EVs treated with Tween20 (P1379; Merk, Sigma-Aldrich); CFSE fluorescence (B-D): FITCH positive events (marking CFSE positive EVs) (B); FITCH positive events (marking CFSE positive EVs) following treatment with Tween20.

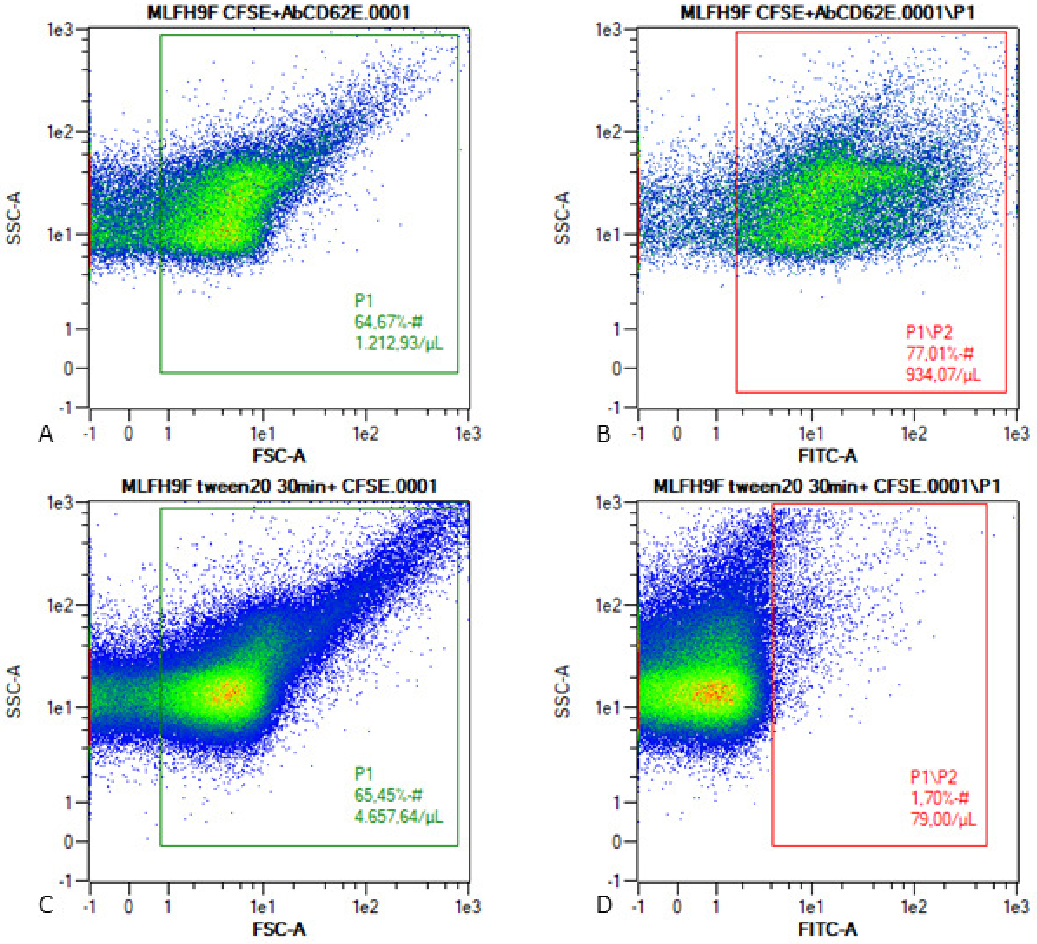

Supplement: Supplementary file 1 [file ijms-20-03669-s001.pdf]
